# The Hypoxia-Adenosine Link during Myocardial Ischemia—Reperfusion Injury

**DOI:** 10.3390/biomedicines10081939

**Published:** 2022-08-10

**Authors:** Wei Ruan, Xinxin Ma, In Hyuk Bang, Yafen Liang, Jochen Daniel Muehlschlegel, Kuang-Lei Tsai, Tingting W. Mills, Xiaoyi Yuan, Holger K. Eltzschig

**Affiliations:** 1 Department of Anesthesiology, McGovern Medical School, The University of Texas Health Science Center at Houston, Houston, TX 77030, USA; 2 Department of Anesthesiology, Second Xiangya Hospital, Central South University, Changsha 410011, China; 3 Department of Anesthesiology, Perioperative, and Pain Medicine, Brigham and Women’s Hospital, Harvard Medical School, Boston, MA 02115, USA; 4 Department of Biochemistry and Molecular Biology, McGovern Medical School, The University of Texas Health Science Center at Houston, Houston, TX 77030, USA

**Keywords:** adenosine, hypoxia, CD73, CD39, Adora2a, A2A, A2B, Adora2b, ENT1, ENT2

## Abstract

Despite increasing availability and more successful interventional approaches to restore coronary reperfusion, myocardial ischemia-reperfusion injury is a substantial cause of morbidity and mortality worldwide. During myocardial ischemia, the myocardium becomes profoundly hypoxic, thus causing stabilization of hypoxia-inducible transcription factors (HIF). Stabilization of HIF leads to a transcriptional program that promotes adaptation to hypoxia and cellular survival. Transcriptional consequences of HIF stabilization include increases in extracellular production and signaling effects of adenosine. Extracellular adenosine functions as a signaling molecule via the activation of adenosine receptors. Several studies implicated adenosine signaling in cardioprotection, particularly through the activation of the Adora2a and Adora2b receptors. Adenosine receptor activation can lead to metabolic adaptation to enhance ischemia tolerance or dampen myocardial reperfusion injury via signaling events on immune cells. Many studies highlight that clinical strategies to target the hypoxia-adenosine link could be considered for clinical trials. This could be achieved by using pharmacologic HIF activators or by directly enhancing extracellular adenosine production or signaling as a therapy for patients with acute myocardial infarction, or undergoing cardiac surgery.

## 1. Introduction

Myocardial ischemia-reperfusion injury is most commonly caused by a mechanical obstruction of a coronary artery, for example by a plaque, thromboembolism or vasospasm [1,2,3]. The subsequent restoration of coronary blood flow will cause inflammatory cells to move into the ischemic myocardial tissues, which provides the immunologic cause of cardiac reperfusion injury [4]. Despite the advancement of clinical strategies to achieve earlier and more persistent reperfusion, myocardial ischemia-reperfusion injury continues to be a leading cause of morbidity and mortality in the USA and worldwide [2,3]. In addition, cardioprotection from ischemia-reperfusion is also critical for patients who are undergoing cardiac surgery, since those patients are at risk for myocardial ischemia-reperfusion injury [5,6]. Novel pharmacologic approaches to render the myocardium more resistant to ischemic tissue injury or dampen myocardial inflammation during reperfusion would be highly desirable and are currently areas of intense research.

During myocardial ischemia, the occlusion of a coronary vessel causes profound changes in metabolic supply and demand within the area that is perfused by the specific artery (so-called “area at risk”). Due to the limited supply of metabolites and oxygen from the bloodstream, the area of risk becomes profoundly hypoxic, thus leading to the stabilization of hypoxia-inducible transcription factors (HIF) [7,8,9,10]. Stabilization of HIF activates a transcriptional program leading to the increased production of the extracellular signaling molecule adenosine [11,12]. These transcriptional changes include enhanced extracellular production of adenosine from precursor nucleotides [13,14,15,16], increased levels and signaling events through extracellular adenosine receptors [17,18], attenuated uptake of extracellular adenosine via adenosine transporters [19,20,21], and the attenuated metabolism of adenosine [22]. Adenosine exerts its cardioprotective effects during ischemia-reperfusion injury through multiple actions, such as vasodilation to increase blood flow and oxygen, decreasing myocardial oxygen consumption, preserving endothelial cell function, and attenuating inflammation. Taken together, studies on the interdependence of hypoxia and adenosine identified an adaptive transcriptional program under the control of HIF that is geared towards promoting extracellular adenosine signaling events on multiple levels. In fact, this molecular link creates multiple opportunities for pharmacologic interventions, including HIF activators, enhancers of extracellular adenosine signaling events, adenosine uptake or metabolism inhibitors, or the use of specific adenosine receptor agonists. Therefore, we will review the mechanism of HIF stabilization during myocardial ischemia-reperfusion injury, its impact on cardiac adenosine metabolism and signaling, and eventually discuss therapeutic opportunities that present themselves through the hypoxia-adenosine link for the treatment or prevention of cardiac injury.

## 2. Hypoxia-Inducible Transcription Factors (HIF) Are Stabilized during Myocardial Ischemia and Provide Cardioprotection

During myocardial ischemia, the cardiac tissues become profoundly hypoxic. This is caused by an attenuated supply of oxygen and metabolites to the area at risk by the occluded coronary artery. Several previous studies have shown that even very short episodes of myocardial ischemia (as short as only 5 min) are associated with the stabilization of HIF [23]. These transcription factors were discovered in the early 1990s in studies of the erythropoietin promoter [24,25,26], a discovery that was subsequently awarded the Nobel Prize in 2019 [27]. HIF are heterodimeric transcription factors with a constitutively expressed beta unit (HIF1B) [28]. In contrast, the alpha unit (HIF1A or HIF2A) is substantially regulated on the post-translational level [29,30,31,32]. During normal oxygen availability, HIF1A/HIF2A are targeted for proteasomal degradation through a molecular pathway that involves oxygen-sensing HIF prolyl hydroxylases (PHD1, PHD2, or PHD3) [33,34,35,36]. PHDs use oxygen as a co-factor to promote hydroxylation of a conserved prolyl-residue with the HIF1A/HIF2A subunit, which subsequently promotes binding of the Von-Hippel-Lindau gene product, polyubiquitination, and proteasomal degradation [31,37,38,39]. However, if oxygen levels fall, PHDs are functionally inactivated. In addition, other metabolic changes in the microenvironment [32,40,41], or oxygen-independent mechanisms of PHD inhibition, have been demonstrated previously (e.g., elevations of succinate levels) [42,43]. These changes in metabolic supply and demand lead to the stabilization of HIF1A or HIF2A, which form a transcriptionally active complex with the HIF1B subunit [44]. This transcriptionally active complex can bind to hypoxia-response elements (HREs) within the promoter region of hypoxia-responsive genes, and promote changes in the transcription rate of the specific gene products. Famous HIF target genes include, for example, erythropoietin or vascular endothelial growth factor. However, studies in genetic models show that approximately 570 genes are transcriptionally altered by the activity of HIF, and most likely more than that [45]. In many instances, HIF binding to HREs will cause transcriptional increases for specific gene products [46,47], but, very frequently, this can also cause repression of a specific gene product [21,45,48,49]. Repression of a specific gene product by HIF is often related to the induction of HIF-dependent microRNAs (miRNAs), which promote the subsequent repression of an indirect HIF target gene [50,51]. For example, a recent study demonstrated that HIF-dependent induction of miRNA miR122 causes repression of PHD1 as an indirect HIF1A-target gene [51].

Many studies on the consequences of HIF stabilization during acute myocardial ischemia-reperfusion injury highlight the protective functions of HIF. These studies include evidence that both HIF1A or HIF2A stabilization can have cardioprotective functions, but most likely involve different tissue-compartments and different hypoxia-dependent target genes [52,53,54,55,56]. Similarly, pharmacologic studies using small-molecular inhibitors of PHDs (PHD inhibitors) demonstrate that pre-treatment approaches are associated with attenuated myocardial ischemia-reperfusion injury [23]. Importantly, orally available HIF activators have recently been used in phase 3 clinical trials for the treatment of renal anemia. These studies showed that HIF activator treatment is at least equally potent to promote hemoglobin levels through the induction of erythropoietin as compared to treatment with recombinant erythropoietin [57,58,59,60]. These pharmacologic HIF activators have rarely been explored in clinical trials for cardioprotection. However, there is strong experimental evidence that those compounds (e.g., vadadustat or roxadustat) could potentially be used to attenuate myocardial ischemia-reperfusion injury in patients with acute myocardial infarction (MI) or for cardioprotection during cardiac surgery [31].

## 3. Role of HIF in Regulating Adenosine Signaling during Myocardial Ischemia-Reperfusion Injury

Several previous studies have proposed linkages between hypoxia, HIF, and extracellular adenosine signaling as a means to providing tissue-adaptation, or to dampen hypoxia-driven inflammation [61,62,63]. In the extracellular compartment, adenosine is generated from precursor nucleotides, such as ATP or ADP [64,65,66,67]. Once adenosine is generated, it can signal through four distinct adenosine receptors, including the adenosine A_1_ receptor (ADORA1), the adenosine A_2_A receptor (ADORA2A), the adenosine A_2_B receptor (ADORA2B), and the adenosine A_3_ receptor (ADORA3) [68,69]. These G-protein coupled receptors have all been implicated in cardio-adaptive responses [53]. For example, the Adora1 is known to mediate the heart-rate slowing effects of intravenous adenosine, used for the treatment of supraventricular tachycardia [70]. In particular, the Adora2a and the Adora2b have been shown to dampen inflammatory responses [71,72,73,74]. For example, Adora2a signaling has been discovered on polymorphonuclear neutrophils (PMNs) [75] and contributes to attenuated inflammatory responses [76,77]. The subsequent uptake of adenosine from the extracellular compartment [49,78], and metabolism to inosine [13,79,80,81] or AMP is implicated in terminating extracellular adenosine signaling. In the next section, we will discuss studies on how HIF-dependent alterations of gene transcription can alter extracellular adenosine signaling during myocardial injury, and how these responses have functional implications on cardioprotection during myocardial ischemia-reperfusion injury.

### 3.1. Impact of Hypoxia-Signaling on the Production of Extracellular Adenosine

During conditions of hypoxia, inflammation, or cellular stress, different cells release nucleotides, particularly in the form of ATP or ADP. For example, ATP can be released from inflammatory cells through specific molecular pathways [16,82,83,84,85,86,87]. The extracellular release of ADP has been described extensively from platelets [68]. ATP or ADP can function as precursor molecules for the extracellular production of adenosine. This process is a two-step, enzymatically controlled pathway. As the first step, the ectonucleotidase CD39 converts extracellular ATP or ADP to AMP [88]. Studies in gene-targeted mice for *cd39* (*cd39*^−/−^ mice) [89] show that these mice experience larger myocardial infarct sizes in the context of diminished levels of AMP and adenosine [90]. Moreover, *cd39*^−/−^ mice are not protected by ischemic preconditioning, where one or more preceding cycles of myocardial ischemia are associated with the attenuated size of injury [90,91,92]. Importantly, several studies demonstrate that the transcript and protein levels, and also the enzymatic function of CD39 are increased during ischemia, inflammation, or hypoxia [15,90,93,94,95,96]. Studies on the transcriptional mechanism controlling CD39 expression during limited oxygen availability link the increased CD39 levels to transcriptional control of Sp1 [97,98].

**Figure 1:**
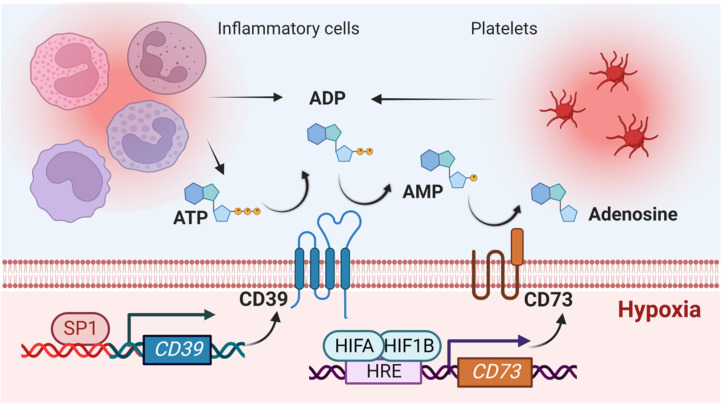
Hypoxia increases extracellular adenosine during myocardial ischemia. In the context of hypoxia, different cell types such as inflammatory cells and platelets release large amounts of adenine nucleotides (particularly ATP or ADP). The ectonucleotidases CD39 and CD73 convert ADP/ATP to AMP and AMP to adenosine, respectively. Therefore, the level of extracellular adenosine during hypoxia or inflammation critically depends on the expression level and enzymatic activity of CD39 and CD73. Hypoxia promotes the induction of CD39 expression through SP1 signaling, and of CD73 expression through binding of the transcription factor hypoxia-inducible factor HIF1A to a hypoxia-response element (HRE) within the CD73 promoter. ATP: adenosine triphosphate; ADP: adenosine diphosphate; AMP: adenosine monophosphate.

The second step for the extracellular production of adenosine is under the control of the ecto-5′-nucleotidase CD73 [99]. This enzyme promotes the extracellular conversion of AMP to adenosine and can be considered as a “pace-maker” for extracellular adenosine generation. Similar to *cd39*^−/−^ mice, gene-targeted mice for *cd73* [100] experience increased myocardial injury and are not protected by ischemic preconditioning [101]. As would be expected based on its enzymatic function, *cd73*^−/−^ mice experience attenuated concentrations of cardiac adenosine in conjunction with elevated cardiac AMP concentrations during myocardial injury [101]. Moreover, transcript, protein, and functional levels of CD73 are increased under hypoxia [101]. Several studies link these increases to a transcriptional program under the control of HIF1A. Studies with transcription factor binding assays and promoter constructs had demonstrated that CD73 is a classic HIF target gene and implicate HIF-dependent induction of CD73 in hypoxia-adaptive responses [93,94,100]. When exposed to myocardial ischemia-reperfusion, *cd73*^−/−^ mice experience larger infarct sizes and higher elevations of cardiac injury markers (troponin I) compared to control animals [101]. Subsequent studies during myocardial injury demonstrate that the protective effects of pharmacologic HIF activator treatment is attenuated in *cd73*^−/−^ mice [23], thereby directly implicating HIF-dependent CD73 regulation in cardioprotection. Together, these studies indicate that during myocardial ischemia, hypoxia signaling through Sp1 and HIF1A coordinate the transcriptional induction of CD39 and CD73, which leads to the increased production of extracellular adenosine and thereby contributes to attenuated myocardial infarct sizes (Figure 1).

### 3.2. Role of HIF in Coordinating Extracellular Adenosine Signaling during Myocardial Ischemia-Reperfusion Injury

**Figure 2:**
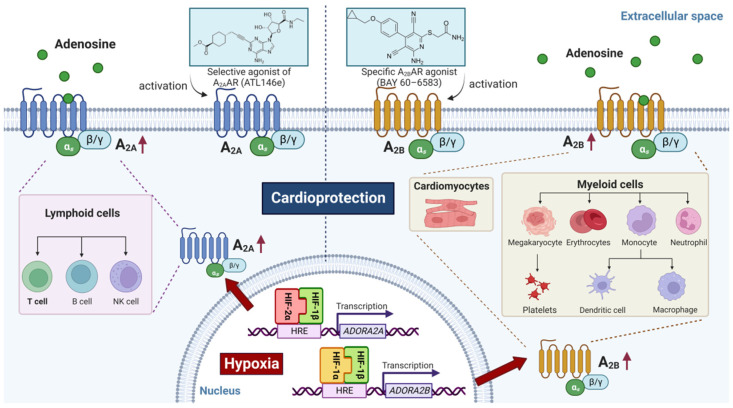
HIF protects against myocardial ischemia-reperfusion injury through the modulation of adenosine receptor signaling events. Adenosine receptors belong to the G protein-coupled receptor family and are composed of different subunits: the Gs alpha subunits (Gαs) and the beta-gamma subunit complex (Gβ/γ). The adenosine receptors Adora2a and Adora2b have been identified as target genes of HIF. Under hypoxic conditions, Adora2a and Adora2b are transcriptionally induced by HIF2A and HIF1A, respectively. Activation of these receptors with their specific agonists showed reduced infarct size in murine models of myocardial ischemia-reperfusion injury, suggesting their role in mediating the cardioprotective effects of HIF. The cardioprotection provided is associated with the activation of Adora2a signaling on lymphocytes and Adora2b signaling on myeloid cells and cardiomyocytes. The red arrowhead denotes upregulation. A2_A_: Adenosine A2a Receptor. A2_B_: Adenosine A2b Receptor.

As described above, myocardial ischemia-reperfusion injury is associated with increased production of extracellular adenosine. Adenosine acts on four different receptor subtypes, including Adora1, Adora2a, Adora2b, and Adora3. All these receptors have been implicated in providing cardioprotection [101,102,103,104]. However, only the Adora2a and the Adora2b are transcriptionally regulated by HIF. They are highly expressed on a variety of different cellular sources, for example on cells of the innate immune system [96,105,106], erythrocytes [18,20], cardiac myocytes [56], stromal or epithelial cells [107,108,109,110], regulatory T-cells [111,112,113], and other immune cells [114]. Several previous studies have shown that the Adora2b promoter contains an HRE and can be directly induced by HIF1A during conditions of hypoxia [115], inflammation [108,116], or during myocardial ischemia-reperfusion injury [23,56]. Similarly, the Adora2a has been previously identified as a target for hypoxia-signaling through the HIF2A isoform [117]. Studies in murine models of myocardial ischemia-reperfusion injury implicate both Adora2a and Adora2b in cardioprotection from ischemia-reperfusion. For example, murine studies demonstrate that infarct size-reducing effects of treatment with an Adora2a agonist are linked to Adora2a signaling on bone-marrow-derived T or B lymphocytes [118], which were subsequently identified to be most likely CD4+ T cells [119]. Similar to the Adora2a, several studies implicate the Adora2b in cardioprotection from ischemia-reperfusion injury. For example, *Adora2b*^−/−^ mice are not protected by ischemic preconditioning and exhibit larger myocardial infarct sizes [101]. Moreover, treatment with a specific agonist for the Adora2b is associated with a significant reduction in infarct sizes in murine [101] or rat [120] models of myocardial ischemia-reperfusion injury. Studies using treatment approaches with the pharmacologic HIF activator dimethyloxalylglycine (DMOG) demonstrate abolished cardioprotection by this treatment in *Adora2b*^−/−^ mice, thereby directly linking HIF1A and Adora2b signaling during cardioprotection [23]. Studies on the cellular source of the Adora2b receptor implicate myeloid-dependent Adora2b signaling in cardioprotection from ischemia-reperfusion injury [121,122]. Other studies suggest that Adora2b signaling on cardiac myocytes or inflammatory cells can interface with the stabilization of circadian rhythm signaling molecules, thereby contributing to the circadian oscillation of myocardial injury [53,56,123,124,125]. In addition, a recent study demonstrated a regulatory function for Adora2b signaling in promoting epicardial stromal cells′ HIF stabilization after myocardial infarction as an additional crosstalk between Adora2b and HIF implicated in cardioprotection after myocardial infarction [109]. Taken together, these findings demonstrate HIF-dependent control of adenosine receptor expression and signaling in attenuating myocardial injury during ischemia-reperfusion (Figure 2).

### 3.3. HIF-Dependent Promotion of Alternative Adenosine Receptor Activation

Several studies implicate the neuronal guidance molecule netrin-1 [126] in alternative mechanisms of adenosine receptor activation, particularly for the Adora2b [127,128]. Netrin-1 was discovered as a neuronal guidance molecule. Its function was originally described as netrin-1 secreted from cells of the floor plate of the mammalian embryonic neural tube [129,130,131]. Its secretion sets up a circumferential gradient of netrin-1, which in some instances attracts or in other instances repels other axons to the ventral midline [126]. Receptors for secreted netrin-1 include, for example, the receptor DCC (deleted in colorectal cancer) [132] and the UNC5 homologs (UNC5A, B, C, and D) [133] and neogenin-1 [134]. Importantly, the profound ability of the netrin-1 in guiding the repulse or outgrowth of neuronal cells makes it an ideal candidate molecule for the coordination of inflammatory cell migration [135].

**Figure 3:**
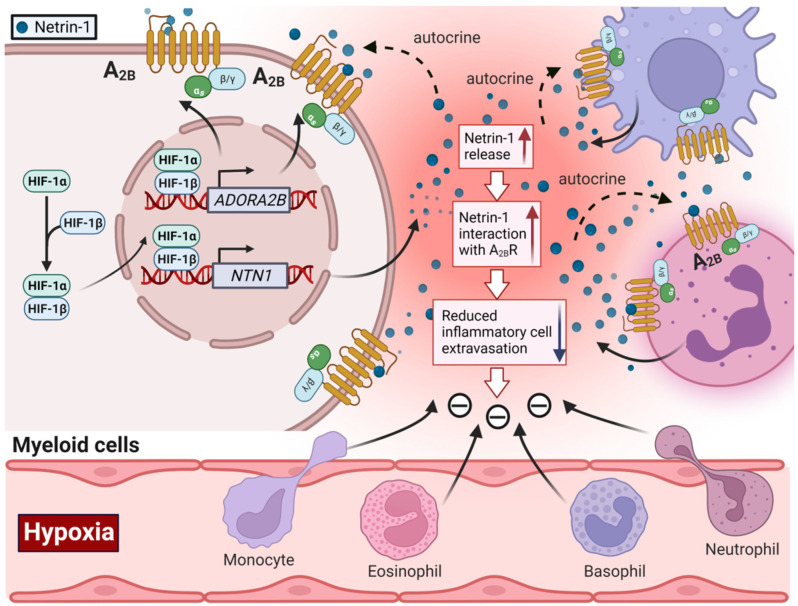
HIF coordinates alternative adenosine receptor signaling via increasing netrin-1 expression and signaling through Adora2b. During myocardial reperfusion injury, different types of inflammatory cells, such as neutrophils, monocytes, etc. infiltrate into the myocardial tissue. This further exacerbates tissue hypoxia and tissue damage. During reperfusion, the transcript and protein levels of Netrin-1 are robustly increased in patients with myocardial ischemia and in mice with myocardial IR injury. The increased expression of netrin-1 is mediated by HIF1A activity, which can bind to an HRE within the *Netrin-1* promoter. The increased release of netrin-1 enhances Adora2b signaling by interacting with myeloid Adora2b in an autocrine manner, dampens the accumulation of inflammatory cells, and ultimately mediates cardioprotection against IR injury. The red arrowhead denotes increase, and the dark blue arrowhead denotes decrease. A2_B_: Adenosine A2b Receptor. *NTN1*: Netrin-1.

Studies utilizing a two-hybrid screen of a human brain cDNA library discovered a previously unreported interaction of netrin-1 with the Adora2b adenosine receptor [136]. Several studies using genetic and pharmacologic approaches demonstrate that netrin-1 can function to promote Adora2b signaling during inflammatory conditions outside of the brain, including acute lung injury [47,128,137,138], inflammatory peritonitis [127], intestinal inflammation [139,140], inflammatory kidney disease [141], corneal wound healing [142], and also myocardial ischemia-reperfusion injury [143]. However, one study found inconsistent results by showing that the Adora2b is actually not expressed in neurons, and is functionally not required for commissural axon guidance in the context of netrin-1 signaling [144]. At present, it is not well understood how netrin-1 and the Adora2b interact, including the possibility that netrin-1 could directly bind to Adora2b as its ligand, a role of netrin-1-dependent enhancement of extracellular adenosine levels, or indirect effects of netrin-1 by binding to a classic netrin-1 receptor and enhancing intracellular signaling cascades under the control of the Adora2b. A recent study found that netrin-1 levels were up-regulated in samples of patients who experienced myocardial ischemia-reperfusion injury [143]. Subsequent studies in mice with deletion of netrin-1 in the myeloid lineage (*Ntn1^loxp/loxp^* LyzM Cre+ mice) revealed selectively larger infarct sizes and higher troponin levels, while other mouse lines with conditional deletion of netrin-1 in other tissues didn’t experience a similar phenotype. Importantly, treatment studies with recombinant netrin-1 demonstrated that the interaction of netrin-1 with myeloid-dependent Adora2b signaling is critical in this pathway, suggesting an autocrine signaling pathway [143]. Previous studies have found that the promoter of netrin-1 contains an HRE and that HIF1A binding to the netrin-1 promoter dramatically increases netrin-1 expression of transcript and protein levels [145]. Subsequent studies in myeloid cells confirmed that finding [146], including recent studies showing that *Hif1a*-deficient myeloid cells fail to induce netrin during injury [47]. In conjunction with these studies, it is conceivable that hypoxia-signaling coordinates netrin-1 and Adora2b signaling in an autocrine loop where neutrophil-derived netrin-1 attenuates myocardial injury through signaling events on Adora2b receptors expressed on myeloid cells of the innate immune system (Figure 3).

### 3.4. Impact of HIF Signaling on Extracellular Adenosine Uptake and Metabolism

**Figure 4:**
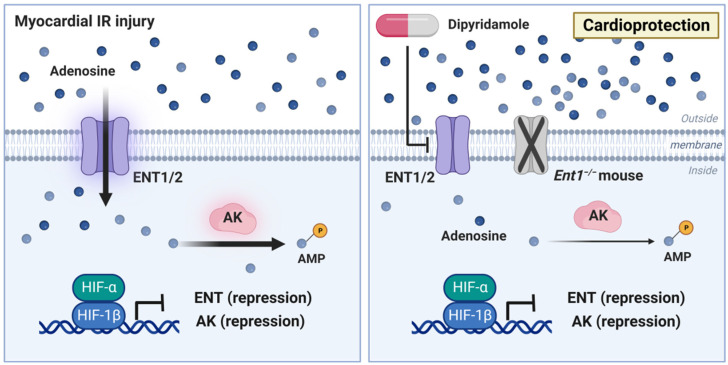
HIF contributes to attenuated adenosine uptake, reduced adenosine metabolism and concomitant cardioprotection during myocardial ischemia-reperfusion injury. Equilibrative nucleoside transporters (ENTs) regulate the uptake of adenosine from the extracellular towards the intracellular compartment where the major routes of adenosine removal is based on phosphorylation to AMP via adenosine kinase, thereby modulating adenosine levels. During myocardial ischemia-reperfusion injury, HIF transcriptionally represses ENT1, ENT2 and adenosine kinase, leading to elevated extracellular adenosine levels. The inhibition of ENTs in mice with dipyridamole or global deletion of *Ent1* showed decreased intracellular adenosine uptake and increased extracellular adenosine levels, ultimately exerting cardioprotective effects. These indicate the contribution of HIF-dependent repression of ENTs to adenosine-mediated cardioprotection. ENT: equilibrative nucleoside transporter; AK: adenosine kinase.

Previous studies have implicated HIF in modulating extracellular adenosine uptake and metabolism. In this context, the consequences of HIF transcriptional activity function towards attenuating extracellular adenosine uptake and intracellular metabolism, thereby enhancing extracellular adenosine signaling events. Adenosine signaling is terminated through equilibrative nucleoside transporters (ENTs), particularly ENT1 and ENT2 [21,78,147]. Those are channels that allow the bidirectional flow of adenosine across the cell membrane following its gradient. Extracellular production of adenosine is dramatically increased and the gradient for adenosine is directed from the extracellular compartment towards the intracellular side during ischemia-reperfusion injury. Therefore, deletion or inhibition of adenosine transporters with an ENT inhibitor such as dipyridamole will result in increased extracellular adenosine levels. Due to its function as an ENT inhibitor, dipyridamole treatment has been used clinically for many decades during pharmacologic stress echocardiography, where it increases coronary adenosine levels, and can unmask coronary artery stenosis [148,149]. Importantly, for the hypoxia-adenosine link during myocardial injury, previous studies have shown that HIF functions to repress both ENT1 and ENT2 during conditions of hypoxia or inflammation, and thereby functions to increase extracellular adenosine levels [17,19,20,21,108,150]. Interestingly, mice with global deletion of *Ent1* experience elevated plasma levels of adenosine, which can contribute to cardioprotection [151]. However, the individual contributions of ENT1 versus ENT2 during myocardial ischemia-reperfusion injury have not been addressed, for example by using genetic murine models. Nevertheless, global inhibition of ENTs with dipyridamole has been implicated in cardioprotection from ischemia-reperfusion injury [152,153]. Taken together, these studies highlight the likelihood that HIF-dependent repression of ENTs contributes to cardioprotection from ischemia-reperfusion injury. However, it would be important to define the individual contributions of ENT1 versus ENT2, as well as their tissue-specific functions and adenosine receptor signaling events in experimental studies of myocardial ischemia-reperfusion injury (Figure 4).

In addition to the repression of ENT1 and ENT2, HIF1A has also been shown to repress a key metabolic process in intracellular adenosine metabolism. Adenosine can be metabolized intracellularly to AMP by the adenosine kinase (AK). Studies on hypoxia responses of AK demonstrate that hypoxia is associated with attenuated transcript and protein levels of AK. Moreover, studies in genetic models directly implicate HIF1A in its repression and demonstrate increased adenosine responses with AK repression [22]. Several studies implicate this pathway in cardioprotection. For example, experimental studies in rats treated with the AK inhibitor iodotubercidin demonstrate attenuated myocardial infarct sizes [154]. Together, these studies indicate the likelihood that HIF1A-dependent repression of AK contributes to adenosine-dependent cardio- protection from ischemia-reperfusion injury (Figure 4).

## 4. HIF-Dependent Cardioprotection beyond Purinergic Signaling Events

**Figure 5:**
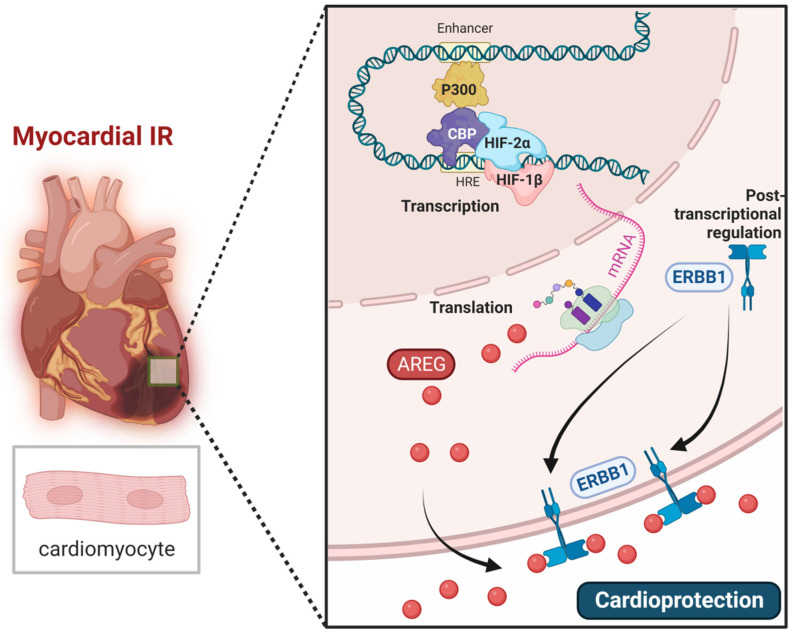
HIF2A induces AREG signaling in cardiac myocytes to provide cardioprotection. HIF2A contributes to cardioprotection during myocardial IR injury. The epithelial growth factor amphiregulin (AREG) has been identified as one of the target genes of HIF2A, which is significantly induced at both mRNA and protein levels in cardiomyocytes during hypoxia. HIF2A was also found to increase the expression of AREG receptor ERBB1 at the post-transcriptional level. These findings indicate HIF2A protects against myocardial IR injury through AREG signaling. ERBB: Epidermal growth factor receptor.

In addition to the cardioprotective functions of HIF1A, several studies revealed that HIF2A also contributes to cardioprotection from ischemia-reperfusion injury. A head-to-head comparison of mice with genetic deletion of *Hif1a* or *Hif2a* in cardiac myocytes revealed larger infarct sizes in *Hif2a^loxp/loxp^* Myosin Cre+ mice compared to Myosin Cre+ controls, whereas there was essentially no difference in infarct sizes between *Hif1a^loxp/loxp^* Myosin Cre+ mice and controls [52]. A subsequent microarray screen for HIF2A targets revealed the epidermal growth hormone amphiregulin (AREG) as the most differentially regulated gene [52]. The epidermal growth factor receptor (EGFR or ErbB1) ligand AREG has been identified to induce activation of the survival kinases Akt in the myocardium to protect against ischemia-reperfusion injury [52]. Previous studies had shown that AREG can be induced by hypoxia, independent of HIF1A [155,156,157]. Indeed, mice with global deletion of *Areg* or mice with myocyte-specific deletion of the Areg-receptor *ErbB1* (*ErbB1^loxp/loxp^* Myosin Cre+) demonstrated increased susceptibility to myocardial ischemia-reperfusion injury [52,54]. Genetic studies in mice with *Hif2a* deletion confirmed the regulatory function for HIF2A for the transcriptional induction of *Areg*. Interestingly, HIF2A was also found to be critical for the induction of the AREG receptor ERBB1; however, this was independent of a transcriptional role of HIF2A [54]. Together, those findings demonstrate the cardioprotective functions of HIF2A expressed in cardiac myocytes by coordinating the induction of AREG and signaling through the ERBB1 receptor (Figure 5), independent of purinergic signaling events.

In contrast, the cardioprotective effects of HIF1A signaling have been suggested in other tissue compartments than myocytes, for example in vascular endothelial cells. Mice with *Hif1a* deletion specifically in vascular endothelial cells were not protected from ischemic preconditioning [158,159]. Moreover, previous studies have implicated HIF1A in mediating the effects of remote ischemic preconditioning [160]. This is an experimental strategy where short repetitive episodes of ischemia to an arm or a leg provide organ protection to the heart or the kidneys from a subsequent ischemic injury [161,162] and have been applied successfully in randomized trials of patients undergoing major surgery [163,164]. Experimental studies demonstrate that remote ischemic preconditioning leads to the stabilization of HIF1A, and subsequent induction of IL-10 as HIF target genes. Cardiac IL-10 signaling is subsequently responsible for the observed cardioprotection [160]. Together, these studies demonstrate that there are multiple functions of HIF1A and HIF2A to orchestrate cardioprotection. While HIF-dependent enhancement of extracellular adenosine signaling is central to its role in cardioprotection, there have also been pathways described that highlight HIF-dependent cardioprotection outside of purinergic signaling events.

## 5. Potential Therapeutic Approaches

Various drugs targeting the different steps of the HIF-adenosine link have been developed for myocardial protection. A summary of some of the published clinical trials and ongoing clinical trials on these medications are presented in Table 1 and Table 2.

### 5.1. HIF Activators

Since the advent of a new generation of HIF activators, such as PHD inhibitors, many of them have finished phase 3 clinical trials and are currently applying for FDA approval for the treatment of anemia in patients with chronic kidney disease. For example, in phase III studies, roxadustat has been shown to increase hemoglobin levels and reduce cholesterol levels in chronic kidney disease patients with or without dialysis [59,60]. Vadadustat also underwent phase III clinical trials, showing an improved iron metabolism and anemia in patients undergoing dialysis [57,165]. Two other orally available PHD inhibitors, daprodustat and molidustat, have also completed phase III clinical trials for the treatment of anemia associated with chronic kidney disease [166,167,168]. Although most previous clinical research on these drugs has mainly focused on treating kidney disease, animal studies have also demonstrated myocardial protection after PHD inhibitor treatment in rodent models of myocardial ischemia-reperfusion injury or heart failure [169,170,171,172,173]. In addition, PHD inhibitors are orally available and have shown favorable short-term safety profiles that would make them ideal for the treatment of myocardial ischemia-reperfusion injury for prophylactic treatments of patients undergoing cardiac surgery. For example, a phase II clinical trial (ROXAMI, NCT04803864) on the efficacy and safety of roxadustat in the treatment of patients with acute myocardial infarction is currently in the stage of recruitment. Our anticipation is that more of these compounds will soon be trialed for cardioprotection in patients.

### 5.2. Adenosine

Adenosine plays an important role in ischemia-reperfusion injury by improving post-ischemic ventricular function, reducing neutrophil activity, and limiting myocardial necrosis and apoptosis [174]. Moreover, adenosine is crucial for ischemic preconditioning- mediated cardiac protection in animal models by decreasing myocytes apoptosis after the reperfusion [175]. Large-scale clinical trials have proven the efficacy of adenosine infusion in reducing myocardial infarction size in patients experiencing acute MI [176,177,178,179,180]. The Acute Myocardial Infarction Study with Adenosine (AMISTAD) trial found that adenosine treatment resulted in a 33% relative reduction in infarct size, with a more profound beneficial effect in patients with anterior wall infarction, although no reduction in the composite endpoint (e.g., death, reinfarction, shock, congestive heart failure or stroke) was observed [176]. The AMISTAD-II trial, which was designed as a follow-up trial of the AMISTAD-I trial to focus on anterior wall ST-elevated patients, did not find adenosine infusion to improve clinical outcomes. However, high-dose adenosine infusion significantly reduced infarct size (infarct size: 11% in the high-dose group and 27% in the placebo group; *p* = 0.023) [177]. In post-doc analysis of the AMISTAD-II trial, early adenosine infusion with reperfusion therapy improved survival and the six-month composite outcome [178] (Table 1). The Attenuation by Adenosine of Cardiac Complications (ATTACC) trial did not demonstrate that low-dose adenosine improved left ventricular function at discharge in patients with acute myocardial infarction receiving thrombolysis, but suggested a potential benefit on long-term survival [179] (Table 1).

In addition to patients with acute myocardial infarction, adenosine has also been evaluated in patients undergoing cardiac surgery. It was used either as an adjunct to intermittent blood cardioplegia or as an intra-aortic infusion before the release of aortic cross-clamp [181,182,183,184], and has been shown to be cardioprotective in some clinical studies (Table 1). Despite favorable trial results, the clinical utility of systemic adenosine is limited by its ultra-short intravascular half-life (<1 s) and its undesirable peripheral hemodynamic side effects such as bradycardia and hypotension. Therefore, a more selective adenosine activator will be highly valuable in providing myocardial protection and avoiding side effects of adenosine at the same time.

### 5.3. Adenosine Receptor Agonists

Neladenoson bialanate is a partial adenosine A1 receptor agonist. Preclinical studies have found that this medication could provide potential cardioprotection by improving mitochondrial function, preventing ventricular remodeling, and reducing fibrosis, thereby preventing ischemic injury [185]. There are two randomized, double-blind, placebo-controlled, dose-finding Phase 2b trials investigating the effects of short-term neladenoson treatment on cardiac structure, function or exercise capacity in patients with heart failure. However, no significant beneficial effects were found [186,187] (Table 1).

Selective A2_A_ receptor agonists are also investigated as a therapeutic approach for cardiovascular diseases. The adenosine A2_A_ receptor agonist regadenoson (Lexiscan; Astellas Pharma Inc., Deerfield, Illinois, U.S.) is a commonly used agent for myocardial perfusion imaging studies. The clinical utilization of this medication for myocardial protection has not been validated.

Methotrexate, originally used as an anti-inflammatory drug for the treatment of rheumatoid arthritis, has received attention in recent years for its anti-atherosclerotic effects by increasing adenosine release and activating A2_A_ receptors [188]. The cardiovascular beneficial effect of methotrexate was initially found through several large retrospective studies. Micha et al. [189], in a meta-analysis of 66,334 patients, found that methotrexate at a median dose of 13–15 mg/week improved cardiovascular outcomes in patients with systemic inflammation. Another meta-analysis by Roubille et al. [190] found that patients with rheumatoid arthritis, psoriasis, or psoriatic arthritis on anti-rheumatic drugs treated with methotrexate have a reduced risk of cardiovascular events. The additional study indicated that methotrexate reduces cardiovascular related death mortality in patients with rheumatoid arthritis [191]. Despite these promising findings, in a phase III clinical trial in patients with stable coronary artery disease and Type 2 diabetes or metabolic syndrome (CIRT), low-dose methotrexate failed to reduce the incidence of cardiovascular events to meet the primary endpoint [192]. However, this trial was stopped early because low-dose methotrexate failed to reduce levels of inflammatory mediators and the incidence of cardiovascular events was similar to the placebo group (Table 1). Whether a higher dose of methotrexate could provide myocardial protection in prospective RCT still needs to be determined.

**Table 1:** Summary of the cardiovascular outcomes in selected clinical trials of drugs targeting different steps of the adenosine pathway.

Studied Drug	Published Year	Author	Trial Name	Patient Population	Sample Size	Intervention Assignments	Outcome
Adenosine	1999	Mahhafey et al. [176]	Acute Myocardial Infarction Study of Adenosine (AMISTAD I)	Patient with AMI undergoing thrombolytic therapy	236	Adenosine or placebo (saline) infusion at 70 µg/kg/min for 3 h within 6 h of MI onset.	Adenosine infusion resulted in a 33% less infarct size compared with placebo.
	2005	Ross et al. [177]	AMISTAD-II	Patients with acute anterior STEMI receiving thrombolysis or primary angioplasty	2118	Infusion of adenosine at 50 or 70 µg/kg/min or placebo for 3 h within 6 h of MI, starting within 15 min before fibrinolysis or percutaneous intervention.	High-dose (70 μg/kg/min) adenosine infusion significantly reduced infarct size (placebo group vs. high-dose group: 27% vs. 11%).
	2006	Kloner et al. [178]	Post-hoc analysis of AMISTAD-II	Patients with acute anterior STEMI	2118	Infusion of adenosine at 50 or 70 µg/kg/min or placebo for 3 h.	In patients receiving early reperfusion therapy (within 3.17 h after MI onset), adenosine infusion significantly reduced 1-month and 6-month mortality and incidence of composite clinical endpoints (death, new onset CHF and re-hospitalization for heart failure) at 6 months.
	2003	Quintana et al. [179]	Attenuation by Adenosine of Cardiac Complications (ATTACC) study	Patients with acute STEMI receiving thrombolysis	608	Adenosine or placebo (saline) infusions at 10 µg/kg/min for 6 h at the start of thrombolysis.	Adenosine infusion did not significantly improve measurements of left ventricular function when assessed by echocardiography before hospital discharge. However, after 12 months of follow-up, adenosine treatment appeared to be associated with a lower risk of all-cause and cardiovascular mortality (about 4% reduction).
	2014	Garcia-Dorado et al. [180]	Myocardial Protection with Adenosine During Primary Percutaneous Coronary Intervention in Pts With STEMI (PROMISE)	Patients with STEMI receiving percutaneous coronary intervention (PCI) within 6 h of symptom onset	201	Intracoronary infusion of 10mL saline with or without 4.5 mg adenosine immediately prior to PCI.	Adenosine treatment before PCI did not show a beneficial effect on infarct size limitation. However, it might benefit patients receiving early PCI after symptom onset (less than 200 min) by reducing infarct size and improving recovery of LVEF after MI.
	1999	Mentzer et al. [181]	*N*/*A*	Patients undergoing CABG surgery	253	Cold blood cardioplegia, or cardioplegia containing 500 μM or 2 mM adenosine.	High-dose adenosine treatment was associated with a lower rate of perioperative myocardial infarction and adverse cardiac events, and showed a trend toward lower dopamine doses.
	2018	Ammar et al. [184]	*N/A*	Patients undergoing CABG surgery	60	Adenosine infusion (150 µg/kg/min) for 10 min into the aortic root, starting 10 min before aortic cross-clamp removal.	Adenosine postconditioning group showed better cardiac function indices, lower cardiac enzyme levels, lower incidence of arrhythmia, less inotropic drug consumption, and shorter ventilation time and ICU stay.
Neladenoson bialanate (partial adenosine A_1_-receptor agonist)	2019	Voors et al. [186]	A Trial to Study Neladenoson Bialanate Over 20 Weeks in Patients with Chronic Heart Failure with Reduced Ejection Fraction (PANTHEON)	Patients with chronic heart failure with reduced ejection fraction (HFrEF)	427	Neladenoson bialanate (5, 10, 20, 30, and 40 mg per day) or placebo over 20 weeks.	In patients with chronic HFrEF, neladenoson bialanate did not show a dose-dependent beneficial effect on cardiac structure and function, cardiac biomarkers, or major adverse cardiac events (cardiovascular death, hospitalization or emergency visits for HF). However, a dose-dependent decrease in renal function was observed.
	2019	Shah et al. [187]	A Trial to Study Neladenoson Bialanate Over 20 Weeks in Patients with Chronic Heart Failure with Preserved Ejection Fraction (PANACHE)	Patients with heart failure with preserved ejection fraction (HFpEF)	305	Neladenoson bialanate (5, 10, 20, 30, and 40 mg per day) or placebo over 20 weeks.	Neladenoson did not show a dose-dependent improvement in exercise capacity (changes in 6-min walk test results) in patients with chronic HFpEF.
Methotrexate	2019	Ridker et al. [192]	Cardiovascular Inflammation Reduction Trial (CIRT)	Patients with stable coronary artery disease (MI or multivessel coronary disease) and Type 2 diabetes or metabolic syndrome	4786	Low-dose methotrexate (15 to 20 mg/week) or placebo.	Low-dose methotrexate did not reduce inflammatory markers levels and cardiovascular events compared with placebo.
	2009	Moreira et al. [193]	Methotrexate Therapy on the Physical Capacity of Patients with Ischemic Heart Failure (METIS Trial)	Patients with ischemic chronic heart failure	50	Methotrexate (7.5 mg/week) or placebo, plus folic acid (5 mg/week), for 12 weeks.	For patients receiving methotrexate, their NYHA score showed an improving trend, but no significant change in 6-min walk test results.

CABG: coronary artery bypass grafting; AMI: acute myocardial infarction; MI: myocardial infarction; STEMI: ST-elevation myocardial infarction; CHF: congestive heart failure; PCI: percutaneous coronary intervention; LVEF: left ventricular ejection fraction; *N*/*A*: not available, NYHA score: New York Heart Association score.

### 5.4. Adenosine Reuptake (ENT) Inhibitor

Equilibrative nucleoside transporter (ENT) inhibitors could serve as potential therapeutics for heart protection by potentiating the protective effects of adenosine. However, currently, there are no clinical trials designed to investigate ENT inhibitors in myocardial infarction. Only one study has attempted to assess the diagnostic and prognostic value of serum Netrin-1 levels in patients undergoing coronary angiography for acute coronary syndromes (Table 2). Future trials focusing on myocardial protection of ENT inhibitors will hopefully shed some light on this promising therapy.

**Table 2:** Ongoing clinical trials targeting the Adenosine pathway for myocardial protection.

Studied Drug	Trial Name	Clinical Trials. Gov Identifier	Patient Population	Purpose of Study
Adenosine	The Effect of Adenosine on Myocardial Protection in Intermittent Warm Blood Cardioplegia	NCT02681913	Patients presenting for mini-invasive mitral valve surgery	To investigate the cardioprotective effects of adenosine enriched cardioplegia in patients undergoing minimally invasive mitral valve surgery.
	Adenosine’s Effect on STunning Resolution in Acute Myocardial Infarction	NCT05014061	Patients with acute STEMI	To assess the effect of 6-h adenosine infusion started before revascularization on the recovery of myocardial akinesia and cardiac function at 48 h in patients with STEMI.
Netrin-1	The Role of Netrin-1 in Acute Coronary Syndrome (ACS-NETRİN-1)	NCT04027127	Patients diagnosed with acute coronary syndrome (ACS) and received coronary angiography	To determine the effect of serum Netrin-1 levels on diagnosis and prognosis in patients presenting to emergency department with ACS.

STEMI: ST-elevation myocardial infarction; ACS: acute coronary syndrome.

## 6. Summary and Future Perspectives

Purinergic signaling events through the activation of extracellular adenosine receptors have long been implicated in cardioprotection from ischemia-reperfusion injury [152,194,195,196,197]. More recent studies using mice with the genetic deletion of adenosine receptors globally, or in individual tissue compartments, have provided additional insight into mechanisms of adenosine-dependent cardioprotection. Moreover, many of these studies were able to link purine metabolism and signaling with the activity of hypoxia-signaling and highlight regulatory functions of HIF in coordinating adenosine-mediated cardioprotection. We are now at a stage where multiple pharmacologic tools are available to modulate the hypoxia-adenosine link for the treatment or prevention of myocardial ischemia-reperfusion injury. These strategies include the use of orally available HIF activators, adenosine receptor agonists, or adenosine transport inhibitors. We anticipated that clinical trials in patients with myocardial infarction or in patients undergoing cardiac surgery will help to bring those pharmacologic interventions from the research laboratory to the patient’s bedside.

## Data Availability

Not applicable.
